# MODULATING OXIDATIVE STRESS AND INFLAMMATION: SODIUM BUTYRATE’S THERAPEUTIC PROMISE IN EXPERIMENTAL COLITIS

**DOI:** 10.1590/S0004-2803.24612025-129

**Published:** 2026-05-25

**Authors:** Najib Muaamer Faed MURSHED, Sushma SWAROOPA, Samah Saleh Ahmed AL-AWADHI, Sharmila Kameyanda POONACHA, Caren D’SOUZA, Kishan Prasad HOSAPATNA LAXMINARAYANA

**Affiliations:** 1 Nitte (Deemed to be University), KS Hegde Medical Academy, Department of Pathology, Deralakatte, Mangaluru, Karnataka, India.; 2 Nitte (Deemed to be University), KS Hegde Medical Academy, Central Research Laboratory, Deralakatte, Mangaluru, Karnataka, India.; 3 Nitte (Deemed to be University), KS Hegde Medical Academy, Department of General Surgery, Deralakatte, Mangaluru, Karnataka, India.

**Keywords:** Sodium Butyrate, ulcerative colitis, oxidative stress, inflammatory cytokines, dss-induced colitis, Butirato de sódio, colite ulcerativa, estresse oxidativo, citocinas inflamatórias, colite induzida por DSS

## Abstract

**Background:**

Oxidative stress and excessive cytokine signalling are central to the pathogenesis of ulcerative colitis (UC). Sodium butyrate (SB) has emerged as a potential therapeutic candidate due to its antioxidant and anti-inflammatory properties.

**Methods:**

UC was induced in Swiss albino mice using dextran sulfate sodium (DSS). Experiment groups included normal control (NC), DSS, DSS + SB, and DSS + 5-ASA. Clinical severity was assessed by the disease activity index (DAI) and colon length. Oxidative stress (MDA, MPO), antioxidants (SOD, GSH), Pro-inflammatory cytokines (TNF-α, IL-6), and histopathological scoring were assessed.

**Results:**

Increased DAI and shortening of the colon length with significantly elevated TNF-α and IL-6 levels and MDA and MPO, along with reduced GSH and SOD levels, which reflect oxidative damage and inflammation induced by DSS administration. Whereas treatment with SB therapy has been shown to lower DAI, preserve colon length, minimize MDA and MPO, restore GSH and SOD activity, and ameliorate cytokine levels, with effects comparable to those of 5ASA.

**Conclusion:**

In DSS-induced colitis, the SB effectively attenuated oxidative stress and pro-inflammatory cytokine responses by restoring redox balance and preserving mucosal architecture. These findings suggest that administering SB as a supplementary treatment may be a viable option for managing UC.

## HIGHLIGHTS

• Oxidative stress and excessive inflammatory cytokine release are central to ulcerative colitis pathogenesis.

• Sodium butyrate exhibits known antioxidant and anti-inflammatory actions in gastrointestinal disorders.

• Sodium butyrate significantly ameliorated DSS-induced colitis by restoring antioxidant defenses and reducing inflammatory cytokines in mice.

• Its therapeutic efficacy was comparable to 5-ASA, highlighting butyrate’s promise as an adjunct therapy for ulcerative colitis.

## RESUMO

**Contexto:**

O estresse oxidativo e a sinalização excessiva de citocinas são centrais na patogênese da colite ulcerativa (CU). O butirato de sódio (BS) tem emergido como um potencial candidato terapêutico devido às suas propriedades antioxidantes e anti-inflamatórias.

**Métodos:**

A CU foi induzida em camundongos albinos suíços utilizando sulfato de dextrano sódico (DSS). Os grupos experimentais incluíram controle normal (CN), DSS, DSS + BS e DSS + 5-ASA. A gravidade clínica foi avaliada pelo índice de atividade da doença (DAI) e pelo comprimento do cólon. Foram avaliados o estresse oxidativo (MDA, MPO), os antioxidantes (SOD, GSH), as citocinas pró-inflamatórias (TNF-α, IL-6) e o escore histopatológico.

**Resultados:**

Observou-se aumento do DAI e encurtamento do cólon, com elevação significativa de TNF-α e IL-6, MDA e MPO, acompanhados de redução de GSH e SOD, refletindo dano oxidativo e inflamação induzidos pelo DSS. O tratamento com BS reduziu o DAI, preservou o comprimento do cólon, diminuiu MDA e MPO, restaurou a atividade de GSH e SOD e melhorou os níveis de citocinas, com efeitos comparáveis aos do 5-ASA.

**Conclusão:**

Na colite induzida por DSS, o BS atenuou efetivamente o estresse oxidativo e as respostas de citocinas pró-inflamatórias ao restaurar o equilíbrio redox e preservar a arquitetura da mucosa. Esses achados sugerem que a administração de BS como terapia adjuvante pode ser uma opção viável para o manejo da CU.

## INTRODUCTION

Inflammatory processes within the gastrointestinal tract contribute to a variety of chronic disorders. Among these, inflammatory bowel disease (IBD), including ulcerative colitis (UC) and Crohn’s disease (CD), is one of the most prevalent and debilitating conditions globally^1^ ^-^ ^4^. IBD is characterized by alternating phases of clinical relapse and remission^1^ ^,^ ^4^. The etiology of IBD remains unknown, while its pathogenesis involves a complex interaction with some factors, including environmental factors, gut microbiome, genetics, and immune response, that disturb intestinal homeostasis and perpetuate the inflammatory cascade^1^ ^,^ ^5^ ^,^ ^6^. This chronic intestinal inflammation is profoundly linked with the overproduction of reactive oxygen species (ROS), increasing oxidative stress^4^ ^,^ ^5^ ^,^ ^7^. An imbalance between increased oxidants and decreased antioxidant activity is a hallmark of IBD Pathogenesis^5^. The disrupted intestinal epithelial barrier and increased permeability, commonly observed in IBD, allow the bacterial products to translocate, such as lipopolysaccharides (LPS), which trigger aberrant immune responses and exacerbate inflammation^4^ ^,^ ^6^ ^-^ ^8^. The pro-inflammatory cytokines such as Tumour Necrosis Factor-alpha (TNF-α), Interleukin-6 (IL-6), and Interleukin-1 beta (IL-1β) play pivotal roles in exacerbating tissue damage^6^ ^,^ ^8^ ^-^ ^10^. Oxidative stress is increasingly recognised as a significant characteristic of IBD pathogenesis, contributing to the initiation and perpetuation of mucosal injury^11^.

Monitoring the complex pathology of IBD using specific oxidative stress and inflammation biomarkers. Malondialdehyde (MDA) is a lipid peroxidation product whose elevated levels reflect cellular membrane damage caused by ROS^4^ ^,^ ^7^. Myeloperoxidase (MPO) is an enzyme primarily derived from neutrophils, which are massively infiltrated into the inflamed intestinal mucosa during IBD^5^ ^,^ ^12^, and promotes the generation of hypochlorous acid, exacerbating mucosal inflammation^13^. In contrast, antioxidants, including superoxide dismutase (SOD) and glutathione reductase (GSH), play a protective role. Together, SOD, GSH, and catalase make up the enzymatic antioxidant defense line that scavenges superoxide radicals and protects tissues from oxidative damage^4^ ^,^ ^7^ ^,^ ^12^. GSH is another critical endogenous antioxidant responsible for detoxifying lipid peroxides and playing a vital role in repairing oxidative injury^1^ ^,^ ^7^ ^,^ ^12^. Its depletion is often observed in active IBD^1^ ^,^ ^3^. TNF-α is a key pro-inflammatory cytokine that stimulates the production of ROS and recruits immune cells to the site of inflammation, leading to the disruption of tight junctions in the intestinal barrier^2^ ^,^ ^14^.

**FIGURE 1:**
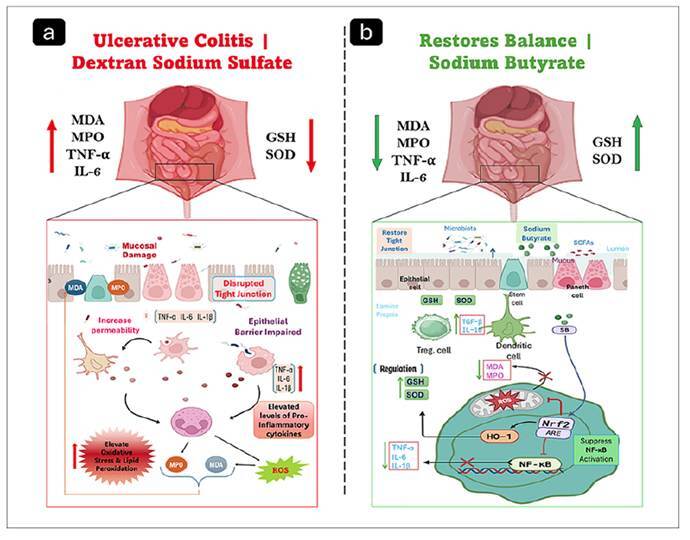
Protective effect of sodium butyrate in DSS-induced ulcerative colitis.

Although extensive research has been conducted on oxidative stress and inflammatory pathways in IBD, there remains a significant gap in demonstrating the correlation between oxidative stress and pro-inflammatory cytokines, TNF-α and IL-6, especially in the context of therapeutic intervention in an experimental model^7^ ^,^ ^15^. While prior studies have measured these markers individually, few have explored how their dynamic relationship is modulated by treatment. Sodium butyrate, a short-chain fatty acid derived from microbial fermentation of dietary fibre, is recognised for its known antioxidant and anti-inflammatory properties and ability to modulate the gut microbiota^2^ ^,^ ^14^. However, its ability to simultaneously regulate oxidative stress pathways and cytokine-mediated inflammation in IBD remains insufficiently explored. Therefore, the present study aims to evaluate the relationship between oxidative stress (MDA, MPO), antioxidants (SOD, GSH), concerning TNF-α levels in a dextran sulfate sodium (DSS)-induced colitis model, and to comprehensively assess the therapeutic impact of sodium butyrate on this complex interplay (Figure 1). Accordingly, the authors hypothesized that sodium butyrate would attenuate DSS-induced colitis primarily through its antioxidant and anti-inflammatory actions, and that improvements in redox balance and cytokine levels would translate into better mucosal preservation.

DSS administration induces colonic injury characterized by oxidative stress, increased pro-inflammatory cytokines, epithelial barrier disruption, and enhanced intestinal permeability (a). Sodium butyrate treatment ameliorates colonic damage by restoring antioxidant defenses, improving barrier integrity, and suppressing inflammatory signaling through activation of the Nrf2/HO-1 pathway and inhibition of NF-κB (b).

## METHODS

### Ethical approval and animals

The experimental protocol was reviewed and approved by the Institutional Animal Ethics Committee (IAEC) of KS Hegde Medical Academy, Nitte (Deemed to be University), Mangalore, India (Ref. KSHEMA/IAEC/2024/10). All possible measures were taken to minimize the number of animals used and to alleviate any pain or discomfort during the experimental procedures.

Swiss albino mice (6-8 weeks old) were obtained from the Institutional Animal House, KS Hegde Medical Academy. All mice were housed under standard conditions (12-hour light/dark cycle, 22±2°C, 50-60% humidity) with free access to standard chow and water.

### Experimental and study design

After one week of acclimatization, mice were divided into four groups, each containing five mice: Normal Control (NC), followed by dextran sulfate sodium (DSS) induction, DSS-induced colitis treated with SB, and DSS-induced colitis treated with 5-aminosalicylic acid (5-ASA). Where the NC group received no treatment. In the IBD-DSS group, colitis was induced using DSS. The SB treatment group was given sodium butyrate for 7 days,andthe5-ASA group received 5-aminosalicylic acid for 7 days after DSS induction (Table 1).

**TABLE 1:** Timeline of the mouse experiment based on groups.

Groups	Day 1-7	Day 8-17 (DSS Induction)	Day 18-24 (Treatment)	Day 25 (Sacrifice)
Group I (NC)	Diet & water	Diet & water	Diet & water	Sacrifice
Group II (DSS)	Diet & water	**DSS administration**	Diet & water	Sacrifice
Group III (DSS + SB)	Diet & water	**DSS administration**	**SB treatment**	Sacrifice
Group IV (DSS + 5-ASA)	Diet & water	**DSS administration**	**5-ASA treatment**	Sacrifice

Colitis was induced in all DSS groups (but not in the N. control group) by administering 2.5% (w/v) DSS (molecular weight 36,000-50,000 Da) in drinking water for 10 consecutive days, with fresh DSS prepared every 2 days. Sodium butyrate was administered at 1 mg/kg body weight and 5-ASA at 50-100 mg/kg body weight, both via oral gavage. Sacrificing the mice under anaesthesia and collecting the colonic and blood samples were processed and stored at −80°C until needed for use.

### Evaluation of disease activity index (DAI) Score

The DAI score was used to assess the intensity of colon damage and clinical progression of colitisthrough daily evaluation of body weight change (%), stool consistency, and rectal bleeding, using a scoring system described by Kim et al.^16^ and illustrated in Table 2.

**TABLE 2:** DAI scoring for clinical progression of colitis.

Parameter	Score 0	Score 1	Score 2	Score 3	Score 4
Weight Loss (%)	0%	1-5%	6-10%	11-20%	>20%
Stool Consistency	Normal	Soft	Loose	Diarrhea	Severe Diarrhea
Rectal Bleeding	None	Slight	Moderate	Severe	Profuse

### Assessment of biochemical markers

### Malondialdehyde (MDA) level

MDA levels were determined in colon tissue homogenates using the thiobarbituric acid reactive substances (TBARS) assay, as described by Ohkawa et al. and Sravathi et al., with minor modifications^17^ ^,^ ^18^. Briefly, 500 µL of the sample was mixed with 1 mL of TCA-TBA-HCl reagent. The mixture was incubated and centrifuged to remove precipitated proteins. The absorbance of the resulting pink chromogen was measured at 535 nm using a UV-Vis spectrophotometer.

### Myeloperoxidase (MPO) activity

MPO activity was measured using a colourimetric method based on the oxidation of 4-aminoantipyrine and phenol by hydrogen peroxide, catalyzed by MPO, to form a chromophore measurable at 510 nm. The sample was prepared as an enzyme extract. The reaction mixture contained 1.5 mL of 4-aminoantipyrine/phenol, 0.4 mL sodium phosphate buffer, 1.0 mL 1.7 mmol/L hydrogen peroxide, and 0.1 mL of the enzyme extract. The change in absorbance was monitored at 510 nm at 30-second intervals for 5 min. The procedure was adapted from established protocols with minor modifications^19^ ^,^ ^20^.

### Reduced glutathione (GSH) level

GSH levels were estimated using the 5,5’-dithiobis-(2-nitrobenzoic acid) (DTNB) method, which is based on the formation of a stable yellow chromophore upon reaction of DTNB with sulfhydryl groups of reduced glutathione^21^. Briefly, onemillilitre of the supernatant was mixed with 1.5 mL of precipitating solution and allowed to stand for 10 min. The mixture was filtered, and 500 µL of the filtrate was combined with 2 mL of 0.3 M disodium hydrogen phosphate solution and 250 µL of freshly prepared DTNB reagent. Absorbance was measured at 412 nm against a reagent blank within 10 min. GSH concentration was calculated from a standard curve prepared using known GSH concentrations and expressed as µg/ml.

### Superoxide dismutase (SOD) activity

SOD activity was determined according to the nitro blue tetrazolium (NBT) reduction method, which measures the inhibition of superoxide anion-mediated reduction of NBT to formazan in the presence of riboflavin and methionine as electron donors. Briefly, the reaction mixture consisted of 0.3 mL of riboflavin, 2.5 mL of methionine, 0.1 mL of NBT, 0.1 mL of the sample, and phosphate buffer to the required volume. After 10 min illumination in a fluorescent light chamber, absorbance was recorded at 560 nm. SOD activity was expressed as Units per Milligram (U/m) protein, where one unit of SOD was defined as the amount causing 50% inhibition of NBT reduction. The total protein concentration was determined using the biuret-based method for normalization. The technique was adapted from previous studies with minor modifications^18^ ^,^ ^22^.

### Cytokine estimation (TNF-α and IL-6, ELISA)

The levels of tumour necrosis factor-alpha (TNF-α) and interleukin-6 (IL-6) were measured in serum using GENLISA Mouse ELISA kits (Krishgen Biosystems, India), following the manufacturer’s instructions. In brief, standards and samples were added to pre-coated microplate wells and incubated at 37°C. After washing, a biotin-conjugated detection antibody was added, followed by streptavidin-horseradish peroxidase and substrate solution. The stop solution halted the reaction, and absorbance was measured using a microplate reader at 450 nm. Cytokine concentrations were calculated and expressed in picograms per millilitre (pg/mL).

### Histopathological analysis

Colon tissues were collected from all experimental groups and immediately fixed in 10% neutral buffered formalin for 24 to 48 hours. The fixed tissues were processed to make the paraffin blocks, whichweresectioned at 4-5 µm, and stained with hematoxylin and eosin (H&E).

Histopathological examination was conducted under a light microscope by a blind pathologist. Colon tissue alterations, including crypt damage, epithelial erosion, goblet cell depletion, and inflammatory cell infiltration, were evaluated. A murine-validated histological scoring system was applied, with scores ranging from 0 (unaffected) to 4 (very severe) as described previously^23^. Representative photomicrographs were captured using the Aperio CS2 digital pathology scanner (Leica Biosystems, Nussloch, Germany) for documentation and comparison across groups.

### Statistical analysis

All data were expressed as mean ± standard deviation (SD). Statistical analyses were performed using GraphPad Prism version 8.0 software (GraphPad Software, CA, United States). Variations among multiple groups were analyzed using one-way analysis of variance (ANOVA) followed by Tukey’s post hoc test for multiple comparisons. A p-value < 0.05 was considered statistically significant.

## RESULT

### Disease activity index scoring

**FIGURE 2:**
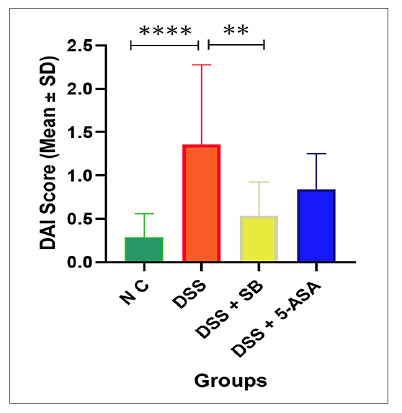
Effect of SB on DAI in DSS-induced colitis.

In the disease activity index, the DSS Group showed a marked increase compared to the NC group (*P*<0.0001). SB treatment significantly lowered DAI relative to the DSS group (*P*<0.01). In contrast, 5-ASA treatment caused only a modest, non-significant reduction (*P*>0.05). Overall, SB effectively alleviated DSS-induced disease activity, demonstrating efficacy comparable to the reference drug 5-ASA (Figure 2).

DAI scores are expressed as mean ± SD (n=5 per group). DSS treatment significantly increased DAI compared with the NC group (****P<0.0001). Sodium butyrate significantly reduced DAI compared with DSS (**P<0.01), while 5-ASA showed only a partial. Statistical significance was determined using one-way ANOVA followed by Tukey’s multiple comparison test.

### Effect of SB on colon length (cm)

**FIGURE 3:**
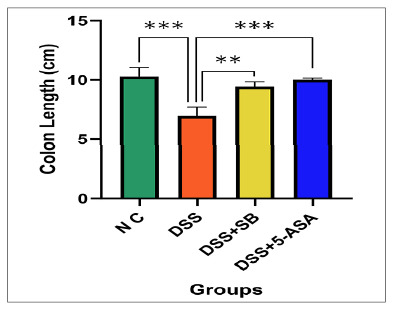
Effect of SB on colon length in DSS-induced colitis.

The DSS group resulted in a marked reduction in colon length compared with the NC group (*P*<0.001). Treatment with SB significantly preserved colon length relative to DSS (*P*<0.01), while 5-ASA produced a similar protective effect (*P*<0.001) (Figure 3).

### Ameli--orative effect of SB on redox balance: attenuation of oxidative stress and promotion of antioxidants.

### Malondialdehyde (MDA) level

**FIGURE 4:**
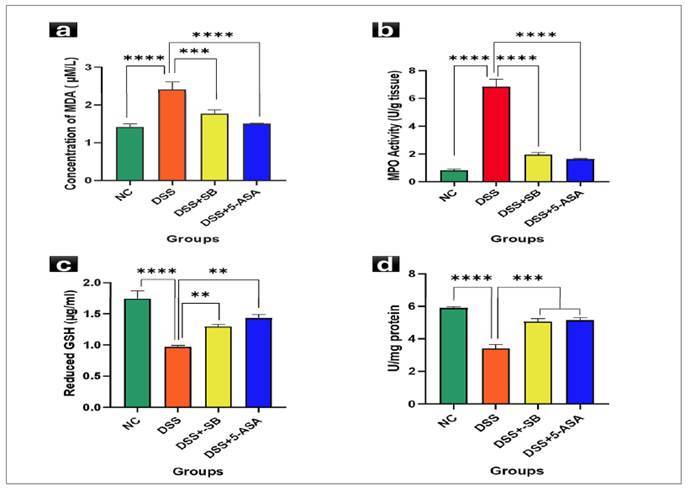
Effect of SB on reducing oxidative stress and enhancing antioxidants.

Malondialdehyde was markedly elevated in the DSS group (2.41±0.19 μM/L) compared with the NC (1.14±0.08 μM/L; *P*<0.0001). DSS + SB group (1.77±0.09 μM/L) significantly attenuated this increase. 5-ASA exerted the most pronounced suppression of MDA (1.51±0.00 μM/L, *P*<0.0001 vs DSS). These findings indicate that SB effectively reduces DSS-induced lipid peroxidation, with efficacy approaching that of 5-ASA (Figure 4A).

DSS markedly increased MDA, which was significantly attenuated by SB treatment and 5-ASA. Data are mean ± SD (n=5). **P<0.001, ***P<0.0001 vs DSS (**A**), MPO activity was significantly elevated in the DSS group, whereas in SB and 5-ASA markedly reduced. Data are mean ± SD (n=5). ****P<0.0001 vs DSS (**B**), DSS depleted GSH, which was significantly restored by SB treatment and 5-ASA. Data are mean ± SD (n=5). *P<0.05, **P<0.01 vs DSS (**C**), and DSS suppressed SOD activity, while SB and 5-ASA restored antioxidant defense. Data are mean ± SD (n=5). ***P<0.001 vs DSS (**D**).

### Myeloperoxidase (MPO) activity

MPO activity was significantly enhanced in the DSS group (6.84±0.16 U/g tissue, *P*<0.0001) relative to controls. The DSS+SB group (1.95±0.22 U/g tissue) and 5-ASA (1.63±0.06 U/g tissue) both markedly reduced MPO activity compared with DSS (all *P*<0.0001). These reductions demonstrate the anti-inflammatory potential of SB in limiting neutrophil-driven tissue injury (Figure 4B).

### Reduced glutathione (GSH) level

Reduced glutathione levels were depleted by DSS (0.97±0.03 µg/ml) compared with control mice (1.17±0.21 µg/ml; *P*<0.0001). The DSS + SB group restored levels moderately (1.29±0.06 µg/ml; *P*=0.03), and 5-ASA demonstrated a more substantial restorative effect (1.43±0.09 µg/ml; *P*=0.003). These results highlight the capacity of sodium butyrate, especially when given after colitis induction, to support endogenous glutathione pools (Figure 4C).

### Superoxide Dismutase (SOD) Activity

The DSS administration group significantly reduced SOD activity (3.42±0.41 U/mg protein) compared to the control group (5.96±0.98 U/mg protein; *P*<0.0001). DSS + SB group (5.05±0.33 U/mg protein, *P*<0.001) restored SOD activity, comparable to 5-ASA (5.15±0.23 U/mg protein). These data confirm that SB preserves enzymatic antioxidant capacity in DSS colitis (Figure 4D).

### Mitigative effect of SB on pro-inflammatory cytokines 

### Tumor necrosis factor-α (TNF-α)

**FIGURE 5:**
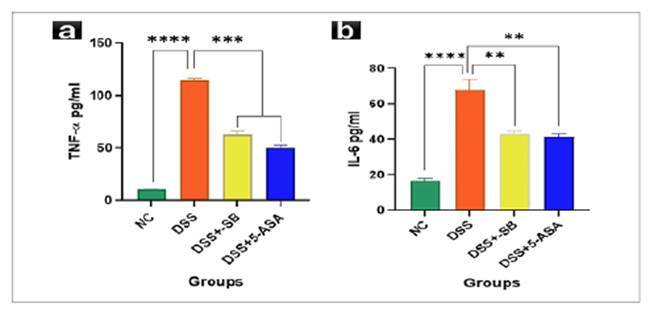
Effect of SB on Pro-inflammatory cytokines.

Serum TNF-α levels were sharply increased following DSS exposure (114.59±2.79 pg/mL) compared to controls (10.52±0.21 pg/mL; *P*<0.0001). DSS + SB group (62.82±4.60 pg/ml) significantly reduced TNF-α, with 5-ASA producing the most marked reduction (50.03±4.00 pg/ml; *P*<0.0001 vs DSS). These data underscore sodium butyrate’s ability to suppress a pivotal pro-inflammatory cytokine in colitis (Figure 5A).

Serum TNF-α levels. DSS markedly elevated TNF-α, which was significantly reduced by SBtreatment and 5-ASA. Data are mean ± SD (n=5). ****P<0.0001 vs DSS (**A**). Serum IL-6 levels. DSS significantly increased IL-6; SB treatment and 5-ASA provided the strongest reductions. Data are mean ± SD (n=5). *P<0.05, **P<0.01 vs DSS (**B**).

### Interleukin-6 (IL-6)

IL-6 levels were further significantly raised in DSS-treated mice (69.79±4.57 pg/ml) relative to controls (16.43±2.30 pg/ml; *P*<0.0001). DSS + SB group (42.80±2.82 pg/ml, *P*<0.01) significantly decreased IL-6, while 5-ASA (41.41±2.97 pg/ml, *P*<0.01) yielded the most substantial reduction. These findings confirm the anti-inflammatory efficacy of SB against DSS-induced elevation of cytokines (Figure 5B).

### Histopathological findings

**FIGURE 6:**
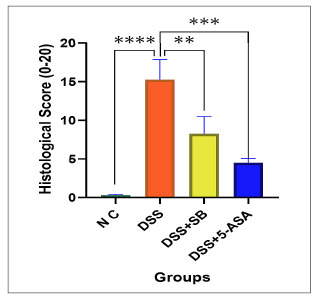
Histological score across groups.

**FIGURE 7:**
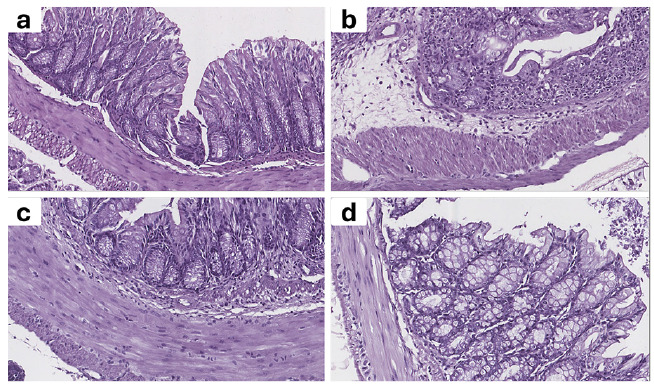
Representative H&E-stained colon sections.

The histological examination of colonic sections revealed significant differences between groups (Figure 6). The NC group had intact mucosal architecture with well-preserved crypts, abundant goblet cells, and no signs of inflammatory infiltrates or ulceration. In contrast, the DSS group exhibited severe histopathological changes, including extensive crypt distortion, epithelial disruption, goblet cell depletion, prominent inflammatory cell infiltration, and mucosal ulceration, which corresponded to a significantly higher composite histological score (15.25±2.63, *P*<0.001 vs control; Figure 7).

DSS administration resulted in a significant increase in histological score compared with NC, whereas both sodium butyrate (DSS+SB) and 5-ASA (DSS+5-ASA) significantly reduced the score. Data are presented as Mean ± SD (n=5 per group). ***P<0.001, **P<0.01 vs DSS

(**A**) NC: intact crypts, abundant goblet cells, no infiltration. (**B**) DSS: severe crypt distortion, epithelial damage, goblet cell loss, and heavy inflammatory infiltration. (**C**) DSS + SB: moderate preservation of mucosal architecture, reduced infiltration, partial goblet cell presence. (**D**) DSS + 5-ASA: near-normal mucosa, minimal infiltration, and preserved goblet cells.

Treatment with SB (DSS+SB) partially ameliorated the DSS-induced damage, as evidenced by moderately preserved crypts, reduced infiltration, and improved goblet cell presence, with a lower composite score (8.25±2.22, *P*<0.01 vs DSS). The 5-ASA-treated group (DSS+5-ASA) demonstrated marked protection, characterized by a near-normal mucosal structure, minimal infiltration, and largely preserved goblet cells, resulting in the lowest composite score among the treated groups (4.50±0.58, *P*<0.001 vs DSS).

Overall, these findings confirm that DSS induces profound colonic injury, while both SB and 5-ASA exert protective effects, with 5-ASA showing the most substantial histological improvement.

### Correlation analysis between oxidative stress, antioxidant status, and cytokines

Pearson’s correlation analysis was performed to explore further the relationship between oxidative stress and inflammatory responses (Table 3). Oxidative stress (MDA, MPO) positively correlated with pro-inflammatory cytokines TNF-α and IL-6 (r=0.85-0.90; *P*<0.001). In contrast, antioxidants (SOD, GSH) displayed strong negative correlations with cytokines (r=-0.75 to -0.92; *P*<0.001). These findings suggest that enhanced oxidative stress is closely linked to cytokine overproduction, whereas preserved antioxidant defences are inversely correlated with inflammatory mediators in DSS-induced colitis.

Table 3: the table demonstrates significant correlations between oxidative stress/antioxidant markers and pro-inflammatory cytokines in DSS-induced colitis mice, with elevated levels of MDA and MPO showing strong positive correlations with TNF-α and IL-6, while higher activities of SOD and concentrations of GSH exhibit strong negative correlations; all associations are statistically significant with *P*<0.001 except for SOD and IL-6, which is significant at *P*=0.001.

**TABLE 3:** Correlation analysis between oxidative stress/antioxidant markers and pro-inflammatory cytokines in DSS-induced colitis.

Parameters	TNF-α (pg/mL)	IL-6 (pg/mL)
MDA (µM/L)	r=0.8652 *P*<0.001	r=0.8496 *P*<0.001
MPO (U/g)	r=0.8981 *P*<0.001	r=0.8477 *P*<0.001
SOD (U/mg)	r=-0.8081 *P*<0.001	r=-0.7511 *P*=0.001
GSH (µg/ml)	r=-0.9190 *P*<0.001	r=-0.8718 *P*<0.001

## DISCUSSION

The results of this current study demonstrate that SB has significant protective effects in DSS-induced colitis, as evidenced by changes in clinical severity, colon morphology, oxidative stress indicators, antioxidants, inflammatory cytokines, and histopathology. Further evidence of the interplay between redox imbalance and inflammation in driving colonic pathology came from correlation analysis. Together, these results suggest that SB has therapeutic potential as a complement to colitis treatment. Because SB treatment in this study was initiated only after DSS exposure, the effects observed represent post-induction therapeutic activity. This timing clarifies that the present findings reflect treatment of established inflammation rather than prevention of colitis development.

The increased DAI score and significant colon shortening, both known indicators, reflected the classical signs of colitis induced by DSS exposure, which is known to cause colon inflammation^16^ ^,^ ^24^. SB treatment significantly attenuated these clinical symptoms, with results equivalent to those of standard colitis treatment (5-ASA). These findings are consistent with a previous study, which indicated that butyrate treatment lowers clinical disease scores and prevents colon shortening in DSS-induced colitis model^25^ ^,^ ^26^. It is frequently used as a substitute for inflammation severity, and the preservation seen with SB confirms its ability to counter the structural effects of DSS injury^6^.

Our data clearly show that DSS disrupts intestinal redox equilibrium, as evidenced by elevated MDA and MPO levels, as well as lower GSH and SOD levels. These Changes align with earlier findings suggesting that central to colitis pathophysiology are oxidative stress, lipid peroxidation, and neutrophil infiltration^27^ ^,^ ^28^. SB treatment helped to rebalance this by enhancing antioxidant capacity while suppressing lipid peroxidation and neutrophil activity. Previous investigations have also shown that^25^ ^,^ ^29^. SB enhances antioxidant activity and reduces oxidative damage in experimental colitis, mechanistically through Nrf2 activation, which promotes antioxidant production and inhibits ROS-generating pathways, thereby attenuating oxidative injury^1^ ^,^ ^3^. 

In UC, the pro-inflammatory cytokines TNF-α and IL-6 are key drivers of mucosal inflammation. As expected, DSS considerably raised inflammatory cytokines, while SB treatment significantly lowered their expression. These results are consistent with reports that butyrate encourages anti-inflammatory immunological responses and reduces cytokine synthesis by inhibiting NF-κB and HDAC activity^29^ ^-^ ^31^. The alleviation of clinical and histological symptoms observed in our investigation most likely resulted from the decrease in TNF-α and IL-6.

Histological examination revealed the degree of DSS-induced damage, including epithelial erosion, crypt damage, goblet cell depletion, and an inflammatory infiltrate, all consistent with DSS colitis^16^ ^-^ ^23^. SB Treatment significantly improved these characteristics, demonstrating maintained epithelial integrity and crypt structure as well as decreased inflammatory infiltration. Semiquantitative scoring supported these findings; SB showed 5-ASA-level protection. These results are consistent with previous studies, which indicate that butyrate supplementation supports mucosal integrity and reduces histopathological severity^25^ ^,^ ^26^.

Correlation analysis showed that oxidative stress markers (MDA and MPO) were positively correlated with pro-inflammatory cytokines (TNF-α and IL-6), while antioxidant parameters (GSH and SOD) were negatively correlated. This highlights the close interplay between oxidative stress and inflammation in the development of the pathogenesis of DSS colitis, thus supporting the notion of a vicious cycle between redox imbalance, injury, and cytokine upregulation^4^ ^,^ ^15^. The ability of SB to affect both redox and immune markers suggests a dual mechanism that interrupts this cycle and restores intestinal homeostasis.

Symbiotic processes are likely the origin of the multifaceted defence provided by SB. SB is an HDAC inhibitor that suppresses pro-inflammatory gene expression and controls chromatin structure^29^. Through receptor-mediated signalling (e.g., GPR109A, FFARs), it promotes anti-inflammatory responses and regulatory T-cell activity^31^. By activating Nrf2-dependent antioxidant pathways, SB enhances endogenous antioxidants^1^ ^,^ ^3^. Moreover, SB supports barrier integrity and goblet cell function, thereby limiting the translocation of luminal antigens^26^. Together, these actions explain the observed improvements across clinical, biochemical, and histological parameters.

SB showed notable efficacy comparable to 5-ASA, a standard therapy for UC^32^. This highlights its translational value both as a supplementary and alternative therapeutic modality. Future studies should focus on maximisingthe availability of butyrate in the colon by adjusting dosage schedules and delivery methods, given that it is rapidly metabolised in the proximal gut.

### Limitations

Although our findings provide strong and compelling preclinical evidence, limitations persist. Though well-known, the DSS paradigm does not cover all facets of human IBD. A direct study of the molecular targets of SB, including specific HDAC isoforms or Nrf2-dependent genes, was not undertaken. Furthermore, the bioavailability of orally administered SB in humans is limited; therefore, tailored formulations or prodrug techniques may be necessary for clinical translation.

## CONCLUSION

This study demonstrates that sodium butyrate attenuates DSS-induced colitis by reducing clinical severity, preserving colon morphology, restoring redox balance, suppressing pro-inflammatory cytokines, and improving histological outcomes. The strong correlations observed between oxidative markers and inflammatory mediators underscore the pivotal role of redox-inflammatory interactions in the pathogenesis of colitis. By exerting dual antioxidant and anti-inflammatory effects comparable to 5-ASA, sodium butyrate emerges as a promising microbiota-derived metabolite with translational potential for inflammatory bowel disease. Future investigations in clinical settings are warranted to validate these findings and explore long-term therapeutic potential.

## Data Availability

Data-available-upon-request.
